# Teachers’ Perceptions of Changes in Their Professional Development as a Result of ICT

**DOI:** 10.3390/jintelligence10040090

**Published:** 2022-10-24

**Authors:** Miguel Ángel Negrín-Medina, Abraham Bernárdez-Gómez, Antonio Portela-Pruaño, Juan José Marrero-Galván

**Affiliations:** 1 Department of Specific Didactics, University of La Laguna, 38200 La Laguna, Spain; 2 Department of Didactics and School Organization, University of Murcia, 30100 Murcia, Spain

**Keywords:** teaching task, change, teaching digital competence, qualitative analysis, ICT, corporate intelligence

## Abstract

The introduction of digital information and communication technologies has influenced many aspects of the teaching profession. In addition to their changing use in the classroom, these technologies have strongly impacted the work and professional development of teachers. In this study, which was framed within the R+D+i project DePrInEd, we analyzed the perceptions, beliefs, opinions, and attitudes of teachers on this subject. We conducted a qualitative study through interviews, with a thematic analysis addressing the duality of technology and change. The results indicated that these produced benefits and created difficulties in the teaching task, with the latter being more demanding. Teachers stated that one of the main difficulties they encountered was related to the evolution of students as a result of technology, both in the school and social contexts. This highlights the risk that its extensive use did not lead to the acquisition of other key competencies, including digital and media competencies, in the school environment. Finally, other dimensions that impacted the corporate intelligence of educational centers included the continuous adaptation and mastery of digital competence required by teachers regarding the changes in their tasks, as well as the excessive bureaucratization that technologies have necessitated.

## 1. Introduction and State of the Art

The teaching and learning processes that occur in educational institutions are affected by the professional experience of the teachers who interact within them, providing learning synergies between collectives (including disagreements and consensus) that help to advance the construction of models for professional teacher learning (Boylan et al. 2018). However, a consensus is lacking on how to measure changes in professional learning and teaching practice and whether these changes influence student achievement (Boylan and Demack 2018). In addition to the above, the generational diversity of teachers impacts the school institution, where older teachers may experience a certain professional anomaly due to institutional support for innovations proposed by younger teachers (Ball 1994, p. 78).

An aspect that generates considerable controversy among teachers is the effective integration of information and communication technologies (ICT) in the classroom, as it highlights the importance of the digital competence development of teachers, as well as their evaluation and promotion among students (Marrero Galván et al. 2021). Digital competencies in teachers are the skills that enable complex digital literacy of students through the teaching and learning process (UNESCO 2018). Moreover, ICT is conditioning the organizational and corporate intelligence of educational centers, as these technologies facilitate access to the information population and have led to online learning emerging as an educational alternative. As such, schools, as social institutions, need to develop the organizational advantages and strengths generated by ICT through resources and procedures that create the appropriate conditions and infrastructure (Rodríguez-Cruz 2015, p. 349) to achieve the construction of intelligent educational organizations. MacGilchrist et al. (2004, p. 15) points out that corporate intelligence in schools consists of those that are able to “synthesize different kinds of knowledge, experience and ideas in order to be confident about current achievements and to have the ability to decide what to do next.” For the development of organizational and corporate intelligence of schools, competent teachers are required to use complex information networks for their application in the teaching and learning process.

Aspects of broad social importance, such as the outbreak of the SARS-CoV-2 virus that triggered the COVID-19 pandemic, have produced profound changes at the global level, leading to substantial alterations in the foundations of educational systems. Thus, for example, steps have been taken to consider new school scenarios in which traditional teaching and learning processes are replaced by new teaching and learning processes based on ICT (Kuric et al. 2021). However, these changes have produced drawbacks that required the digital competence of teachers to be addressed (Cabero-Almenara 2020).

In many cases, these difficulties are associated with generational differences among teachers (Cabrera 2020). This may have contributed to the emergence of negative stereotypes regarding the ability of certain generations of teachers to use ICT, which may indicate a negative self-perception about their technology skills, and is associated with the physical, social, and personal competence decline that occurs with age (Köttl et al. 2021). Jiang et al. (2016) showed that despite the existence of this generational gap regarding ICT, the three generations studied (silent generation, baby boomers, and millennials) showed a need for training in digital skills, such as online safety, but also that this training should be adapted to the circumstances of each generation. In the case of millennials, for example, they do not require the same training as older teachers, since they have grown up with and are trained in the digital language (Prensky 2010).

For teachers who currently have more experience, the use of ICT was not part of the requirements when they entered the profession. However, this skill is required of the new generations of teachers. Therefore, the former is facing a professional deficit that they have had to overcome in the course of their teaching work. This shows that the understanding and development of skills required for the use of digital technologies by teachers of any generation should be present in their continuing education (Kaloyanova and Ivanova 2013). That is, teachers who are digital migrants may experience difficulties with students, which new teachers, who are already considered digital natives, do not. Therefore, teachers of older generations often need more training to meet these challenges. The difference in knowledge between teachers is evidence of a digital gap between them. According to Prensky (2010, p. 6), they use an obsolete language, “the language of the pre-digital age” to instruct a generation that knows the new language perfectly well.

All of these factors seem to hinder the development of the corporate intelligence of educational centers, whose foundation can be developed through cooperative learning in virtual pedagogical ecosystems (Palmero et al. 2016) and facilitating technological teacher updating (Cotino 2021), for example, through personal learning environments (Domínguez 2016). Schools and teachers have been forced to change, not only regarding the use of ICT in the framework of teaching and learning processes, but also in the methods of communicating with students and families. These processes are now often being conducted through the use of social networks (WhatsApp, Facebook, Instagram, etc.) and through school management applications that allow both families and students to access data on curricular progress, attendance, performance, daily work, school services, etc. (Blau and Hameiri 2012; Bouhnik and Deshen 2014; Hadad et al. 2020). In summary, and following Weisberger et al. (2021), educational systems worldwide have been affected by the profound technological development of the digital era in recent decades, which has led to change in schools through the following:

- The integration of ICT in teaching and learning processes, which requires both the migration of existing curricular materials to the new digital environments and the creation of new materials in line with new technology-based methodologies, such as gamification based on virtual reality.
- The development of new online learning communities that directly impact the development of the professional teaching career.
- The aforementioned new forms of communication between teachers, students, and families.

All of this has led recent educational leaders to encourage a real and substantial integration of ICT through reforms in educational systems. An example of this is the Digital Compass 2030, presented by the European Commission, which aims to ensure that technology is at the service of citizens during this decade (European Commission 2021). The key actions include both the “Digital Education Action Plan to boost digital literacy and skills” and the “European Skills Agenda with a strong focus on digital technologies” for Europe’s digital transformation and resilience (European Commission 2020). In 2021, Resolution 2021/C 66/01 of the Council of Europe on a strategic framework for European cooperation in the field of education and training (2021–2030) (DOUE 2021) was also published to digitize European education systems. It provided them with adequate technological infrastructures, as well as introducing technology into the teaching work environment and boosting their digital competence (Alija 2021), in turn creating high-performance digital education ecosystems and educational policies that enable the acquisition of basic digital competencies of citizens at school.

However, the integration of ICT as intended by the European Commission in its digitization policy is not possible without the effective participation of teachers; the idiosyncrasies among groups of teachers can considerably affect this integration. In addition, any intended reform must consider, among other factors, the existing generational diversity (Mohammadi 2019). In a study conducted with early childhood education teachers, Rodríguez et al. (2019) found that the ability of students to learn digital skills seems to be negatively affected by generational differences between teachers, especially regarding generating their own content with a high level of difficulty and being aware of their own training deficit.

In addition to positive and negative emotions regarding the use of ICT in the classroom, Monteiro et al. (2020) outlined myths about the motivation, competencies, and digital skills of older teachers. Similarly, Bjørgen et al. (2021) emphasized the negative perception among teachers that any educational reform that attempts to achieve a paradigm shift in the integration and use of digital technologies in the classroom is unsuccessful, as these programs only add more complexity to the teaching process.

Notably, this study was part of the R+D+i project “Intergenerational Professional Development in Education: Implications in the Professional Initiation of Teachers (DePrInEd),” whose objectives are to generationally characterize teachers (according to their differences and affinities) and identify the interactions between them during the performance of the profession, exploring the perceived impact on learning and improvements in learning processes during their professional careers. As indicated above, the implementation of ICT in the classroom, with its technological evolution and the change it generates in the teaching task, seems to be one of the processes that has the most impact on the different generations of teachers during their professional development. Therefore, some questions arose that we aimed to answer through this study:

- How has ICT affected teachers in the development of their work?
- Do teachers show resilience or weakness in the face of the technological changes in ICT and the development of the new digital competencies required for their work?
- Do teachers feel capable of developing digital competencies with the students of the Z and Alpha Generations, the latter of which, by definition, comprises digital natives?

## 2. Materials and Methods

### 2.1. Objectives

In order to answer these questions, we set the following objectives:

- To analyze the perceptions, beliefs, opinions, and attitudes of teachers about the changes in the teaching task after the infiltration of ICT in schools in recent years.
- To understand the role of technology as a driver of change in the teaching profession and its influence on the development of corporate intelligence.

### 2.2. Design and Sampling

As indicated, we designed this study in the context of the R+D+i DePrInEd project, which is based on a descriptive approach with a mixed (qualitative and quantitative) and multiphase methodology (Portela et al. 2022). We approached the qualitative aspect of our study through semi-structured interviews. This methodology allows for the understanding of participant experiences in a real context, with the researcher accessing their meanings through interaction with the participants (Denzin and Lincoln 2018). Its design corresponds to what was identified by Merriam and Tisdell (2016) as a basic qualitative study. Specifically, it has been used the constructivist-interpretivist approach (Denzin and Lincoln 2018).

The teachers participating in this study were from different areas of non-university education: early childhood, primary, and secondary education (including those who provided vocational training in this group). We conducted a purposeful sampling following the snowball sampling method (Emmel 2013; López-Roldán and Fachelli 2015) while excluding the “professional culture” effect, which is associated with the type of educational center, from the study.

We categorized the participants according to their generational situation into novice (YBT), veteran (MVT), and retired (ORT) teachers. All teachers born after 1990 and with no more than 6 years of teaching experience were considered novice teachers, whereas veteran teachers were those aged 50 years or older and with at least 10 years of experience in non-university educational centers. Regarding retired teachers, only those who reached the age of forced or voluntary retirement were considered, excluding those who retired for health reasons. The decision to include retired teachers was based on the evidence of continuous and even development of professional identity beyond retirement (Shlomo and Oplatka 2020).

The total sample amounted to 60 teachers (20 of each generational stage studied), whose geographical distribution covered various autonomous communities in the Spanish state. At the same time, a homogeneous sample was sought, taking into account the educational stage of the teachers. Thus, 15 teachers were selected from each of the educational stages studied: early childhood education, elementary education, secondary education and vocational training. For this paper, they have been grouped into elementary and secondary education. We adjusted the number of required participants to the sample size suggested in the literature (Sim et al. 2018), including those proposed to reach an adequate level of saturation (Hennink and Kaiser 2022). The most relevant characteristics of the selected participants are listed in Table 1. We invited participants to provide this information in a short electronic questionnaire, which we directly confirmed with them in the interviews.

### 2.3. Data Collection and Analysis

We collected data from semi-structured interviews that addressed the main topics designated in the DePrInEd R+D+i project, including the recurrent and spontaneous perceptions, beliefs, opinions, and attitudes of the participating teachers on ICT. For data collection, we followed the protocol that we established, conducting the interviews by videoconference (recorded with the prior authorization of the participants) due to the COVID-19 pandemic situation.

The protocol for the interviews included a small number of basic questions that allowed for probing and follow-up questions to be introduced as they were conducted. We included the following questions: (1) What is your vision of the teaching profession to which you dedicate yourself (or had dedicated yourself, if the participant was a retired teacher)? (2) Why do/did you have that vision? (3) How have/had you come to see teaching as a profession, and why? (4) With whom do/did you share a vision of the teaching profession, and why?

Subsequently, we transcribed the recordings in full and verbatim. The lengths of the interviews ranged from 28 to 136 min, with the average length being 66.3 min. We conducted the interviews between April and December 2021.

We analyzed the data by combining thematic and comparative analyses (Braun and Clarke 2012, 2022), following the following phases:

- Team familiarization with the data.
- Codebook generation, which involved segmenting meaningful data to identify codes and then defining and exemplifying them.
- Establishing themes of interest, searching for themes to obtain patterns of relationships between data and research questions, and using central categories organized in different clusters of codes with shared traits.
- Themes revision to check their quality by confirming them with the data obtained to confirm, correct, or reject central categories.
- Establishing the final themes, delimiting and connecting them with others, and generating definitions associated with outstanding quotations of the situation described helped to clarify the theme.

We flexibly interpreted the data in accordance with its theoretical foundation through thematic analysis (ICT and change); using comparative analysis, the phases followed tended to overlap and be recursive; therefore, we compared some codes or categories with others, constantly and cyclically (Saldaña 2021).

We conducted the qualitative analysis using ATLAS.ti V22 software (Scientific Software Development GmbH) because, according to Soratto et al. (2020), this program allows for simple visualization of the qualitative information of interest through figures that depict the dynamics of comparison and contrast between codes or categories assigned to the data.

## 3. Results

After reviewing the different topics that could have been related to the study subject, as indicated in phase three of the analysis, we identified two potential topics for review. In order to select these topics, we used ATLAS.ti tools (a table of code co-occurrences^1^) and our analysis. First, we selected the term referring to technologies in the teachers’ discourse, “scope of the ICT” (1.09.12 in the codebook). After searching for other themes that could have been strongly linked to this term, we decided to select the code “change in the task” (1.06.12 in the codebook) because different codes emerged which mentioned one change or another that was linked to ICT. Thus, the discussion presented below is linked to these two central categories, establishing themes of interest in terms of ICTs and change.

As an overview, ICTs have been evolving since they began to be implemented in schools. “In the past, I don’t know, I had that kind of information […] Now, we are lucky that we have the Internet” (D63:8.YBT.Sec[Secondary])^2^. The mention of these ICTs, potentially in any context, signified change, adaptation, novelty, etc. For the teachers, every time a new tool of this type was introduced to a classroom, educational center, or teaching task, it had multiple implications for them. To some extent, we clarified these implications through the following points.

**Figure 1:**
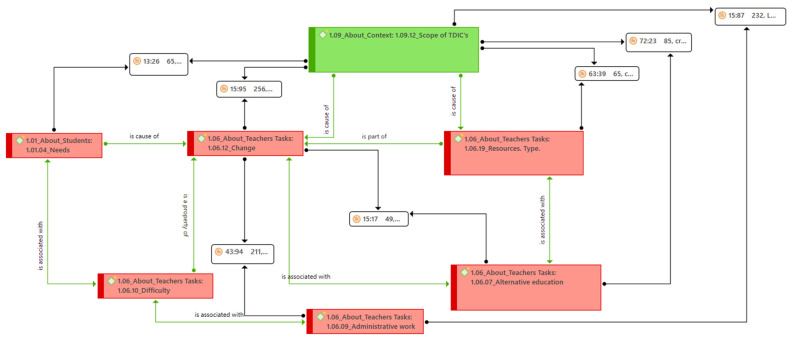
Semantic network of the relationships between the codes associated with the scope of changes brought about by ICTs in teaching tasks.^3^

Figure 1 shows the different results that we present here. Taking the central category selected for the scope of ICT as a basis, the different relationships that emerged with other categories can be observed. Initially, we found that teachers noted the changes produced by various technologies since their implementation. These transformed the tasks which they perform, from methodological processes to administrative tasks in the course of their functions. The introduction of these tools had diverse and beneficial effects, for example, due to the new needs of students who were born with these technologies around them. However, adaptation, with its consequent difficulties, was necessary, both in the use of the mentioned tools and for the characteristics of these students. In addition, the digitization of many teaching tasks has created new loads in the daily work of these students. Examples of this are the digital bureaucratization and the new resources to be used, which were seen as challenges for the professional development of teachers.

In order to further explain the above, we provide a breakdown of the different mentioned categories and what teachers said about each one of them. In addition, we outline the scope of the ICTs considered. Table 2 presents a brief definition of each of the categories we used to help improve reader understanding.

### 3.1. Changing Tasks

As mentioned above, the change in the tasks of teachers over time has been a constant feature in the education field over the last few years. The numerous social demands produced from the end of the twentieth century to the present time have forced the educational field to try to follow the externally marked compass. ICT was one of the main reasons for this dynamism regarding teaching tasks: “changes, especially in terms of technology, in the way of reaching students, and I try to be, to be there, to keep up to date, well, yes, I am very persistent, so I will succeed” (D1:28.MVT.Sec).

The various changes in their activities made adaptation a necessity that required greater daily effort. Adaptation did not exclusively belong to the social sphere because, in the classrooms, we observed how students were experiencing the change in the first person: “Now they have … Those who have more or less, at home, have many options, without even leaving home, to pass the time and not dedicate anything to study. Everyone, most of them, have a computer, have Netflix …” (D64:23.MVT.Sec).

The changes produced by the evolution of students, their dedication to schoolwork, their habits in relation to their studies, and the availability of different tools and resources meant that the teachers needed to consider adaptation from the early stages of education. Moreover, adaptation needed to be a constant that was often not accompanied by the availability of resources in the classroom and/or the evolution of these resources: “We have to make a technological education from the beginning, with the little ones, as we say, but then not to forget it in primary school, we know that many times the computer classrooms are not prepared because otherwise …” (D15:95.ORT.El[Elementary]).

We heard proposals that called for technological training from an early age. Perhaps we can question whether such training should be in technology or for technology, because the teachers had the resources, but not the competence, to use them properly. Thus, the teachers needed to face new difficulties derived from this change, which especially stood out in the change in the objective of the task, namely, the students, and how to reach them: “the only motivation they find is through a screen, and they have not been taught to interact with others, to look for play in others, to look not only for dyadic relationships, but also in small groups, then, this has a great influence” (D69:51.YBT.El).

This lack of motivation and the need to capture the attention of and reach their students caused the teachers to intensely seek a change in one of the issues that was the object of reproduction for decades; technology, among other factors, was forcing the change. One way to promote this change arose from how the teachers developed their teaching.

### 3.2. Alternative Education

In the teaching–learning process, how teachers approach their students and develop their work in the classroom are especially relevant factors. Something common to all the participants in our study was that “the new generations, what they are bringing is new methodologies, new concepts, new technologies” (D70:51.MVT.El). This made the method of approaching students, development in the classroom, and implementing new methodologies in the A/E process dynamic. The classroom was becoming a laboratory in which many ways of keeping students engaged were being tested. This was a tool for establishing a bond of knowledge between teachers and students: “a young teacher, now, with new methods that are closely related to ICTs or something like that, connects more with kids than those of us who are of a different age” (D25:2.MVT.Sec).

Technologies, in addition to providing methods of linking students with their learning and developing closer relationships with them, were how many of the new methodologies currently being developed in the classroom were being used. ICTs were so succinctly introduced that, at present, understanding education without them and without the changes they have produced is difficult. Some of them are relevant in the connection between subjects or the acquisition of new competencies, skills, or ways of understanding reality for students.

*All these things are great for the student, you get them hooked right away and then from there the subjects have to be globalized, everyone has to try to work together, teamwork, collaboration, cooperation is a very important thing.*

*(D15:16.ORT.El)*

Skills, understanding the environment instead of having to memorize why it happens that way; educating, teaching to have critical thinking, which I think before was like: “This is like this and this is how it is” You had to learn that because it was that way” (D72:23.YBT.Sec).

Among these new competencies developed by students as a result of the introduction of technologies in the classroom and their lives was learning management. The autonomy facilitated by ICTs was the force driving a revolution in student access to information.

*I would summarize it in learning to learn, in being able to learn things, to learn themselves […] they have, at the click of a button, at the click of a button, they have all the information. So what they demand is that we provide them with these tools, how they can learn by themselves, being the protagonists of their own learning.*

*(D13:26.YBT.Sec)*

The ease with which the students could find information of interest and the guidance of teachers in the management and channeling of this information made the student the main actor in learning. In addition, the starting point was provided by the students, who were curious to independently learn or solve problems, although always under the guidance of a teacher who understood the importance of this in their daily lives.

*Techniques, and the free and accessible programs that help a lot and support teaching, and I think that in general, practically everyone now has had at least a greater contact with them, at least; and that has favored, yes, that there is more interest and more awareness of how important it is in our daily lives.*

*(D68:42.YBT.Sec)*

One origin of this importance was one of the most perceptible issues: the change that occurred in the teaching, techniques, and programs, i.e., the tools and resources that have been incorporated into educational centers and classrooms. Likewise, the generalization of these and the availability of these resources by teachers has nuances that must be considered.

### 3.3. New Tools and Resources

In Section 3.2, we describe a new way of approaching the teaching task and the methodologies that teachers apply in the classroom, as it relates to two of the main changes that have occurred in recent decades as a result of the introduction of technologies. The know-how that has emerged since the end of the 20th century in relation to ICT changed how skills were developed, a process which had traditionally occurred differently; these skills were adapted to the resources available to teachers and students in the classroom.

*In the classroom they had it as one more tool and material in those areas of activities that children have in the classroom, we could not forget reading and writing, that the child had to read, that the child had to write and although a keyboard—with a keyboard they learn to read and write—of course they learn to read and write.*

*(D15:11.ORT.El)*

However, these new tools and resources were not only being used in schools. The students arrived at educational centers with an amount of knowledge that often surprised the teachers, either because of their sophistication or the level of competence of the student regarding the tools they knew: “Nowadays children, at the age of eleven, already know how to use PowerPoint, Excel, Word, and applications equivalent to other brands or other companies” (D46:30.YBT.Sec).

The new resources available to teachers has changed how they work and perform their activities outside the classroom. The different tools, together with the new methodologies they use, have led to the continuous emergence of many resources. Some tools were traditionally dedicated to other fields, such as business, and are now being used in teaching and learning.

*Now, a lot of people work with these things that are done digitally, some videos … Things that they do … Applications, lots and lots of applications or presentations.*

*(D36:90.MVT.El)*

The vast majority, at least, we all know applications, the typical ones nowadays: Genially, Canva, Plickers, those kinds of things well, we like them, we carry them out (D63:17.YBT.Sec).

Many other teaching-specific tools also appeared, and thus, teachers did not have to spend time developing materials for their classrooms. The different suppliers saw the teachers’ demand for and use of the educational products they provided.

*In Didactalia, there are a lot of interactive maps that have been around for a long time, and for the autonomous communities instead of using the traditional map of a lifetime.*

*(D63:39.YBT.Sec)*

Just as companies focused their business on providing teachers with resources to perform their tasks, administration also sought to take advantage of the different resources provided by technologies. Among them, teachers highlighted issues that they would never have thought of encountering in the classroom that, today, in addition to the simplicity of implementation, help them to deal with the diversity they experience in the classroom.

*eTwinning projects have helped us to get to know other languages, to get to know other children, which is the same thing we have in the classrooms because we often meet children who are Russian, or children who come from Czechoslovakia, from Yugoslavia—excuse me—the new European countries.*

*(D15:17ORT.El)*

Addressing diversity through the technological resources available to teachers was also a method to address other, more urgent issues in their classrooms. The information and knowledge that could be acquired through these resources could positively impact the tackling of educational problems traditionally assigned to specialist teachers.

*Now, we are fortunate that we have the internet, there are a lot of resources at our fingertips and we can have information on how to treat ASD, ADHD, Asperger’s, even if we haven’t been trained on it. But we can have that information, so maybe that will help us.*

*(D63:8.YBT.Sec)*

Thus, the different changes that occurred in the work of teachers also contributed to the work. However, in addition to advantages, certain drawbacks emerged as a result of the introduction of technology to teaching. The main drawback stemmed from the difficulty teachers experienced in facing these changes and the different challenges they constantly faced.

### 3.4. Added Difficulty to a Complex Profession

ICT has introduced new visions, resources, and methodologies, as well as what these entail in the performance of tasks that teachers must perform. However, the teachers we interviewed also identified different issues to be considered in the implementation of these technologies, and that, to some extent, the change increased their daily workloads and presented a challenge in their professional careers. Therefore, some of the most relevant difficulties were adapting to a new context or new working conditions, especially when the teachers were older. In addition, the students had changed, as had the type of bureaucratization that they had to deal with in their work, which were not easy issues for them to solve.

#### 3.4.1. Adapting to Change

Any change requires adaptation, and any adaptation requires an effort, however minimal it may be. With the arrival of ICT in the work of the teachers, they saw how their dedication to ICT increased over time. Many of them described the change that had occurred in the development of their work since they began to practice the profession, and that it was not a simple task: “until computers appeared, because we worked in one way, we studied in one way and from then on we worked in another way” (D25:94.MVT.Sec). These changes occurred so quickly that the teachers felt overwhelmed, which may have damaged or added to the problems and overload to their daily tasks. This, consequently, provoked a rejection of ICT and insecurity in the teachers that they had not previously experienced.

*I see that technologies are eating us, because the truth is that the kids are more … they have much more preparation in this than us and then in a way the technologies force us a little, it conditions us a lot, then almost all the courses we are doing and so on, the technologies are there, the TICs.*

*(D25:52.MVT.Sec)*

The older teachers realized the change was reflected in how different tasks were performed, and that this change was already coming with the new teachers who joined the educational centers: “the current generation is the telematic generation. Everything is based on telematic, computerized development, everything has to go through the computer, they have lost that touch of working with the wrist, of writing” (D12:60.MVT.Sec). This way of working that evolved with technology not only resulted in difficulties in performing various aspects of teaching tasks, but also presented a sense of inevitability. Rejecting change was not an option for teachers: it was a demand that they needed to meet because change was required by the students who were currently in the classrooms.

*If you don’t know how to use a digital whiteboard right now in a classroom, then you’re lost. Children get bored, if what they want is also digital, then you have to adapt to all the media that are available ….*

*(D70:93.MVT.El)*

This change was demanded by the students who were in the classroom and for whom technology is present in their everyday lives. As such, the teachers needed to keep pace with these digital natives.

#### 3.4.2. Students as Digital Natives

The changes that the teachers needed to face were in part related to the context in which they needed to operate. One of the main changes they needed to address was the presence of digital natives in their classrooms. This implies “changes at the level, especially in technology, in the way of reaching the students, and I try to be, to be there, to keep up to date, well, I am very persistent, so I am going to get there” (D1:28.MVT.Sec). This led to difficulties for teachers because of the new tools and adaptation required when teaching if they wanted to apply new methodologies. Technology produced students who were often more immersed in digital environments or with technology than the teachers; therefore, they needed to adapt to them:

*Society, with children whose childhood has nothing to do with ours, then, maybe you can teach them something, maybe more than what we learned, but it will sound like Greek to them, because nowadays everything has changed.*

*(D70:96.MVT.El)*

This was also motivated by the demands and needs that were perceived by teachers in the society of their students: “if you want to educate children for the society we are in today, you have to use the resources they have at home” (D49:21.YBT.El). The students must acquire a set of competencies that will help them to develop in society, and life in general, after school. The context in which students live is digital and technological; therefore, teachers needed to prepare them for the requirements of the future.

*I believe that students are in a kind of transition towards something new. Let me see if I can explain, nowadays the use of new technologies, all this new information society—as I said before—is bringing me new professions, even new communication systems, social networks and other small things.*

*(D13:50.YBT.Sec)*

All of these changes also had negative impacts on the needs of students and the demands of society, because the use of technology was perceived as a cause of other problematic issues faced by schools.

*There are other little things that worry me or other issues that are important and are not very good […] when it comes to writing, writing, the issue of spelling mistakes, the simple fact of how to send an email.*

*(D13:16.YBT.Sec)*

That many of them, the lack of sport, the lack of physical activity, is also noticeable in their motor level […] I see students who have more difficulty at ages where perhaps they should have a greater degree of coordination and it is not so great (D13:17.YBT.Sec).

The use of technology led to changes in students or situations that posed a certain risk. One risk described by many teachers was the lack of effective competencies to develop in society, because student action was focused on the use of technologies without contemplating the use of these technologies to acquire other competencies. They ended up managing technologies from a single perspective. Teachers stated that “I get much more out of those who are natives, who are great, who know how to do a lot of things, but do not know how to apply it to something concrete” (D12:62.MVT.Sec).

#### 3.4.3. Administrative Work

Related to the changes in teaching tasks (mentioned at the beginning of Section 3) is a specific issue that, due to its commonality in the teachers’ discourse, deserves special mention: a form of bureaucratization fostered by technology. An example of this is the implementation of technologies that caused discomfort due to the difficulty involved, and the dedication to tasks that are not educational and, therefore, are outside the teaching field.

Then, it improved a little with the pencil case, where you could put notes and all that, then the evaluation, for example, all the steps, the previous ones we have had in the evaluation, it has also been very hard, the last reforms are useless […] many times it makes it worse because there is so much red tape that if you have to do economic issues, the director is not only a director, he is an administrative director, an executive director, a director of everything, we are teachers who at one stage of our lives are directors (D15:87.ORT.El).

Although technology made it easier to perform certain tasks, the number of tasks multiplied: “Then technologically everything has changed even to give a programming. It does it by standards, I do not know what everything: it has become very bureaucratized no, that change yes” (D43:94.MVT.Sec). Teachers needed to face the bureaucratization of the tasks that were directly related to teaching, as well as those that came about with technology.

*What has changed the most is that, I think the bureaucracy has been increasing, I think you have noticed that with the sites and technology, that everything is technological and that is the application and I don’t know, what mail, WhatsApp, video calls, I don’t know how much. It is all technological.*

*(D43:27.MVT.Sec)*

Thus, most of this technological change was confronted by the teachers. In addition to the students, teachers were the object of change, as they were responsible for bringing the technologies to the classroom to keep pace with the demands of society.

## 4. Discussion and Conclusions

The influence of ICT on the professional development of teachers and how to manage the resulting difficulties or changes were the major focuses of this study. We investigated the changes produced in the teaching task as a result of the recent increase in the use of ICT in the classroom, and approached it from the perspective of considering the role of ICT as a driver of change in the teaching profession. The increase in the use of ICT has involved numerous challenges throughout the professional trajectory of teachers (Davis-Singaravelu 2022). These are the new scenarios in which teachers and schools must advance, establishing a joint vision and action (Azaza et al. 2021). However, ICT has not been a limitation for teachers who have resolved the potential difficulties they have faced and have developed the capacity for the change required in smart educational institutions. As seen through the results, the different teachers described the dynamism and adaptation required to face these different challenges. Thus, some of the dimensions of the corporate intelligence of schools, such as collegial or reflective dimensions (MacGilchrist et al. 2004), have become visible. Fundamentally, these dimensions are visible in two specific areas. The first is the ability of teachers to face the challenges posed by ICTs in a co-collegial manner. This has been facilitated by the existing generational diversity, and, thus, has proved to be beneficial from the perspective of the different generations of teachers. The second is the ability shown by teachers to address the requirements of changing contexts and tasks, which involves observing the different challenges they have faced and acting according to the demands of a profession in which change is a constant throughout their careers. The interviewed teachers considered that the use of ICT had benefits, but also added difficulties, similar to what was reported by Bjørgen et al. (2021) and Monteiro et al. (2020). The teaching task has irrevocably changed over the last few years, constantly evolving to higher demands (Weisberger et al. 2021). This has meant that teachers must develop an increased capacity for reflective intelligence, as they have had to test themselves in the face of new challenges and develop deep learning in the context of ICT.

As such, considerations have emerged for teachers regarding other issues that have transformed during their professional development: the evolution of students and how technology has conditioned their way of relating, their dedication to schoolwork, habits in relation to their studies, and their lack of motivation or the need to capture their attention. These issues have forced teachers to change how they teach, which represents, in turn, an opportunity to establish a knowledge link between teachers and students. This is a fundamental aspect that must be considered to ensure that the actions of teachers throughout their career generate work environments that adapt to the circumstances in which different social demands are encountered. Thus, as school is an intelligent environment, teachers must have the capacity for intelligent learning in the face of change (Davis-Singaravelu 2022; MacGilchrist et al. 2004).

In contrast with the traditional teaching–learning models that hinder the implementation of ICT (Aguiar et al. 2019), teachers who intelligently act in the face of change show commitment to autonomy in learning through the use of ICT, which, in turn, has stimulated a real revolution in the access to information by learners (Pérez et al. 2011). This is an evident sign of pedagogical intelligence, because teachers and schools combine knowledge in a strategy that is aimed toward student involvement. Likewise, the variety and number of resources available have changed how teachers work, although not always with sufficient reflection (Martínez-Sánchez 2003), as well as how they perform their activities outside the classroom (Lozano and Sánchez 2018). Educational innovation is often conceived as linked to the technology of the digital era (Aguiar et al. 2019).

Current students in the Z and Alpha Generations are strongly linked to digital technology, which poses a huge challenge from the viewpoint of educational endeavors. Ziatdinov and Cilliers (2021), in the higher education context, highlighted the influence of networks and social connections and the high perception and ability of Generation Alpha to interpret information as elements that should be considered in future approaches in the teaching–learning process in educational environments. Some researchers found that teachers with different teaching styles are aware of the methodological changes to which they are forced by ICT to adapt for Generations Z and Alpha, especially for the latter, who show a much stronger interest in technologies than previous generations (Nagy and Kölcsey 2017; Tafonao et al. 2020).

However, ICT has also created new difficulties for those in the teaching profession. Thus, regarding teacher adaptation to the change, some veteran teachers indicated that they successfully adapted, but others expressed feeling overwhelmed and relieved that their teaching career was about to end. Different realities exist among digital migrants and their adaptation to new teaching processes. Conversely, novice teachers perceived that this change had not affected them and that no adaptation had been necessary, as they had already achieved digital competence. They have benefited from having the digital language as a native language, as Prensky (2010) points out. Therefore, different beliefs and opinions formed for veteran and novice teachers show a generational difference; however, consensus and a conceptual framework are still lacking (Oh and Reeves 2014). As stated by Monteiro et al. (2020), with sufficient prevention of the generation of myths about digital skills, teachers can develop the competencies and motivation to change their practices.

Another difficulty identified by the interviewed teachers, in line with observations reported by Fernández-Cruz and Fernández-Díaz (2016) and Padilla (2018), is that students are often more immersed in digital environments or technologies than the teachers, which is an aspect that is especially common for older teachers (Han and Nam 2021), because they sometimes do not have sufficient digital competence as a necessary part of the competency–role profile of the modern teacher (Kaloyanova and Ivanova 2013). However, Although teachers are aware of the risk that this entails for the students, because the observed lack of effective competency on the part of students to function in society may be due to their education mainly focusing on the use of technologies, without contemplating using them to acquire other key competencies. Additionally, the extensive use of ICT does not guarantee the acquisition of the digital and media competencies that are required by 21st-century citizenship (Mesquita-Romero et al. 2022). Likewise, the excessive bureaucratization produced by technology, which was initially considered to facilitate and simplify tasks has, on the contrary, multiplied and increased the workload in recent years (Pérez et al. 2011). This has generated discontent and concern among teachers, who express that they do not have time for what they consider to be more important, which could be summarized as improving their training and adequately preparing for their classes.

Notably, we considered the duality of ICT and change in the context of the DePrInEd research project, obtaining results of interest for the teaching profession and the professional development of teachers, but further studies are required. Therefore, we are planning future studies related to school institutions as intelligent contexts in order to determine whether the effect of the implementation of ICT in teaching has been due more to social than pedagogical motivation, as well as to analyze the possible negative effect of ICT on teachers and whether this may lead to burnout.

## Figures and Tables

**Table 1:** Characteristics of participants.

	YBT (n = 20)			MVT (n = 20)		ORT (n = 20)		
	Mean	SD		Mean	SD	Mean		SD
Age (years) ^a^	27.7	2.1		56.2	3	67.9		5.5
Teaching Experience ^b^	2.3	1.7		26.1	7.1	35.6		5.5
	YBT		MVT		ORT		Full sample	
	n	%	n	%	n	%	n	%
School stage								
Elementary ^c^	10	50	10	50	10	50	30	50
Secondary	10	50	10	50	10	50	30	50
Sex								
Female	14	70	16	80	9	45	39	65
Male	6	30	4	20	11	55	21	35

^a^ Age as of 31 December 2021. ^b^ Years of experience when answering the initial electronic questionnaire. ^c^ This category included both early childhood (2nd cycle) and primary education.

**Table 2:** Meanings and number of quotations of the categories used.

Code	Definition	Number of Quotations
1.09_About_Context: 1.09.12_Scope of ICTs	Participant highlights general expansion and penetration of ICT, NNTT, etc.	127
1.06_About_Teachers Tasks: 1.06.12_Change	Participant emphasizes changing or dynamic nature of the task.	169
1.06_About_Teachers Tasks: 1.06.07_Alternative education	Participant associates teaching task with using alternative strategies or methods to those considered “traditional.”	236
1.06_About_Teachers Tasks: 1.06.09_Administrative work	Participant highlights functions associated with bureaucratic activities in the teaching job.	144
1.06_About_Teachers Tasks: 1.06.10_Difficulty	Participant highlights the difficulty, in general, of the task or the problems or difficulties involved in completing it.	174
1.06_About_Teachers Tasks: 1.06.19_Resources. Type.	Participant highlights the relevance of certain resources.	123
1.01_About_Students: 1.01.04_Needs	Participant highlights identifiable needs in the student body or parts of the student body.	235

## Data Availability

Not applicable.

## Notes

1 The code concurrence table establishes the strength of the relationship between two codes using a coefficient indicated in the software. The different relationships established between the different codes were based on a concurrence coefficient relevant to the data analysis. For more information on the program functionalities, please visit https://atlasti.com/manuals-and-documents/atlas.ti_manualwin.v22.pdf/ accessed on 1 July 2022.

2 To reference the quotations extracted from the interviews, we used the referencing system provided by the analysis program. First, we present the digits indicating the document number (D), and then, after the colon, the digits referring to the number of the quotation within the document. In addittion, the generational stage to which the teacher belongs and the educational level at which he/she teaches have been added to each of the quotations.

3 Different elements can be observed in the network. In green the central category analyzed and, in red, the different categories related to the first one or between them. Each of the categories is linked by a specific relationship that makes explicit the type of relationship that these categories maintain (green lines). In addition, since the software does not allow the coefficient of concurrence between two codes to be displayed to show the relationship, we have chosen to show a quote representative of that relationship.
